# Functional Specialization of Skin Dendritic Cell Subsets in Regulating T Cell Responses

**DOI:** 10.3389/fimmu.2015.00534

**Published:** 2015-10-22

**Authors:** Björn E. Clausen, Patrizia Stoitzner

**Affiliations:** ^1^Institute for Molecular Medicine, University Medical Center of the Johannes Gutenberg-University Mainz, Mainz, Germany; ^2^Department of Dermatology and Venereology, Division of Experimental Dermatology, Medical University of Innsbruck, Innsbruck, Austria

**Keywords:** contact hypersensitivity, cross-presentation, dendritic cells, immunotherapy, infectious skin disease, Langerhans cells, Langerin, skin cancer

## Abstract

Dendritic cells (DC) are a heterogeneous family of professional antigen-presenting cells classically recognized as most potent inducers of adaptive immune responses. In this respect, Langerhans cells have long been considered to be prototypic immunogenic DC in the skin. More recently this view has considerably changed. The generation of *in vivo* cell ablation and lineage tracing models revealed the complexity of the skin DC network and, in particular, established the existence of a number of phenotypically distinct Langerin^+^ and negative DC populations in the dermis. Moreover, by now we appreciate that DC also exert important regulatory functions and are required for the maintenance of tolerance toward harmless foreign and self-antigens. This review summarizes our current understanding of the skin-resident DC system in the mouse and discusses emerging concepts on the functional specialization of the different skin DC subsets in regulating T cell responses. Special consideration is given to antigen cross-presentation as well as immune reactions toward contact sensitizers, cutaneous pathogens, and tumors. These studies form the basis for the manipulation of the human counterparts of the murine DC subsets to promote immunity or tolerance for the treatment of human disease.

## Introduction

The skin is the second largest barrier organ to the outside world besides the intestine. As such it is not only exposed to physical stress but also to a wide variety of environmental antigens, including chemicals, commensal bacteria, and pathogens. Hence, the skin immune system must be prepared to detect and discriminate between these diverse antigens and subsequently induce appropriate tolerogenic or protective immune responses. To this aim, the skin contains a heterogeneous population of dendritic cells (DC, from Greek *dendron* “tree”) that represent key regulators of both innate and adaptive immune responses. While skin DC play a critical role in guarding the host against invading pathogens and at the same time limiting collateral tissue damage, they are also associated with the breakdown of peripheral tolerance leading to chronic immune-mediated inflammatory diseases such as allergic contact dermatitis and psoriasis. As essential mediators of cutaneous immune reactions and homeostasis, considerable work has been focused to unravel the origins, phenotypic, and functional differences of the cells of the skin DC network (1–3).

Anatomically, the skin can be divided into an outer epidermis and the underlying dermis, which are separated by a basement membrane. The cell-free basement membrane acts as a mechanical barrier, however, its primary function is to anchor the epithelium (epidermis) to the loose connective tissue (dermis) underneath. The epidermis represents a stratified epithelial layer composed of keratinocytes that generate the water-impermeable *stratum corneum*. The dermis is a cell-poor layer consisting of fibroblasts that produce the extracellular matrix containing proteoglycans and entwined collagen and elastic fibers. Together they enable the skin to resist stretching and tearing forces. In addition to forming the primary physical barrier, keratinocytes also actively contribute to the immunological barrier of the skin. They are equipped with most toll-like receptors (TLR), except TLR7 and TLR8 (4–6). Following TLR triggering and NOD-like receptor (NLR)-mediated inflammasome activation, keratinocytes secrete antimicrobial peptides and many proinflammatory cytokines as well as chemokines for the recruitment of neutrophils. Thereby keratinocytes participate in adaptive immune activation, via inducing DC mobilization and migration to skin-draining lymph nodes (LN), and innate immune modulation (7).

DC can be subdivided into conventional DC and plasmacytoid DC (pDC). Healthy skin contains no or very few pDC (8, 9); they only enter inflamed skin to promote wound healing through type-I interferons (9) or mediate the proinflammatory reaction that develops after TLR7 stimulation, for example, during psoriasis (10). An excellent overview of pDC biology and plasticity has recently been published elsewhere and these cells are not further discussed here (11).

**Figure 1:**
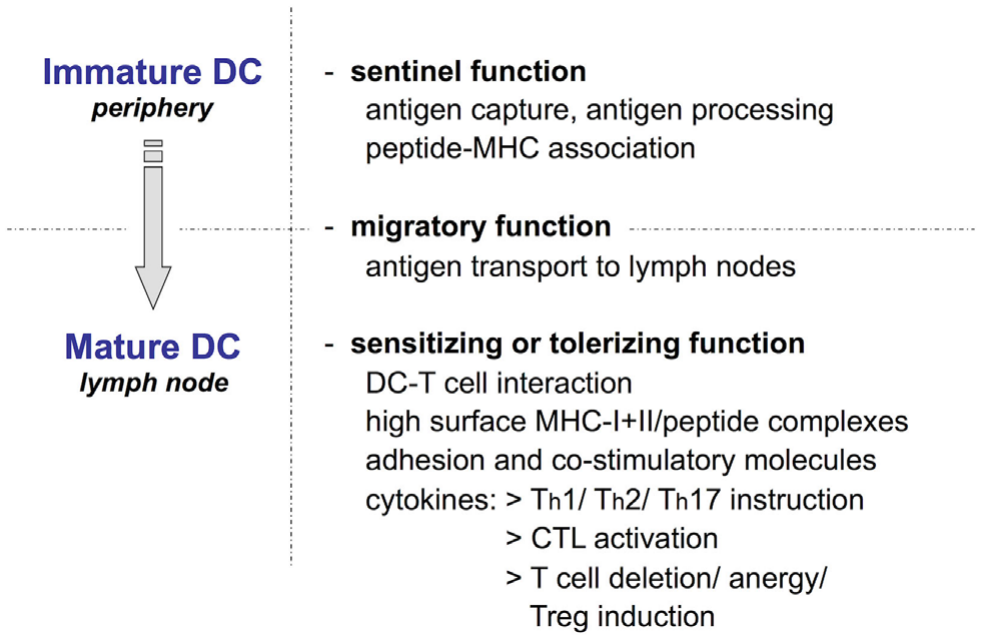
**The Langerhans cell paradigm: Ralph Steinman’s scheme of key dendritic cell functions**. DC, including epidermal LC, exist in two phenotypically and functionally distinct states: as immature cells highly specialized in antigen uptake and processing and as mature cells committed to antigen presentation that activate or tolerize naïve T cells. The two functional programs are connected by DC migration from peripheral tissues to draining LN, which is essential for naïve T cells to encounter their cognate antigen.

In the steady state, the conventional DC residing in the skin are not inactive. Rather as immature cells, they constantly probe their environment for invading pathogens and continuously sample self- and environmental antigens (Figure 1). To this aim, epidermal Langerhans cells (LC) exhibit a unique behavior characterized by rhythmic extension and retraction of their dendrites through intercellular spaces between keratinocytes, which is amplified during inflammation (12). In fact, LC can extend dendrites through tight junctions to survey the skin surface and elicit humoral immunity to antigens that have not yet violated the epidermal barrier, providing preemptive immunity against potentially pathogenic skin microbes (13).

A small fraction of LC and dermal DC undergoes spontaneous maturation through a mechanism that is not yet understood (14). This *homeostatic* or *phenotypic maturation* involves the upregulation of chemokine receptor CCR7, which enables DC migration to the skin-draining LN (15), and in the case of LC downregulation of E-cadherin to detach themselves from the surrounding keratinocytes (16). Moreover, disruption of E-cadherin binding may actively promote a tolerogenic LC phenotype via the release and nuclear localization of β-catenin (17, 18). During their migration to the T cell areas of local LN, the cells upregulate surface expression of MHC/peptide complexes for recognition of and interaction with antigen-specific naïve T cells (Figure 1) (19–22). Upon encounter with potentially autoreactive T cells that have escaped central tolerance or with T cells recognizing peptides derived from innocuous foreign antigens, these DC induce T cell anergy or deletional T cell tolerance (*tolerizing function*) (23–26). In addition, the frequent T cell–DC contacts during T cell scanning of DC in lymphoid organs, i.e., in the absence of cognate antigen, induce a basal activation level in T cells required for rapid responsiveness to subsequent encounters with foreign antigen during inflammation (27).

Pathogen invasion together with proinflammatory signals drive the full *functional maturation* of skin DC. Beyond the homeostatic differentiation program, the cells now also upregulate the expression of costimulatory molecules and, in particular, proinflammatory cytokines. Together these promote clonal expansion of naïve antigen-specific T cells and instruct the T cells to acquire appropriate effector functions specifically tailored to eliminate the invading pathogen (*sensitizing function*) (Figure 1) (19).

In this review, we describe our current understanding of the composition of the skin DC network and summarize the transcription and growth factor requirements for the development of the different skin DC populations. We then discuss the functional specialization of skin DC subsets in the context of allergic and infectious skin disease models, as well as their cross-presentation capacity and their role in skin cancer. Finally, we focus on how this knowledge may be applied to harness skin DC for therapeutic purposes and, to this aim, conclude with a comparison of mouse and human skin DC subsets.

## The Skin-Resident Dendritic Cell Network

**Figure 2:**
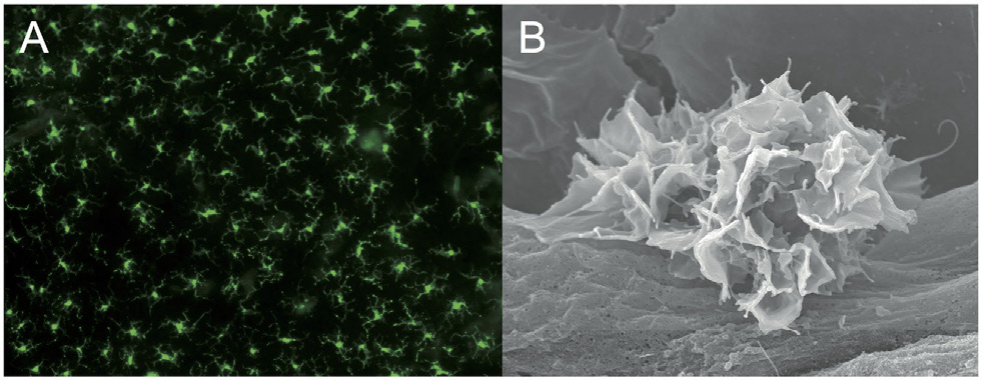
**Langerhans cells: sentinels of the skin**. **(A)** LC network visualized in an epidermal sheet of mouse ear skin with MHC-II antibody staining (green fluorescence) (37). Photograph by courtesy of Julia Ober-Blöbaum and Björn Clausen. **(B)** Scanning electron microscopy of a LC sitting on a keratinocyte (38). Photograph by courtesy of Kristian Pfaller and Patrizia Stoitzner.

After the discovery of DC by Ralph Steinman and Zanvil Cohn in 1973 (28), it was only in 1985 that epidermal LC (Figure 2), first described by Paul Langerhans as “*Nerven der menschlichen Haut*” more than a century before (29), were unequivocally placed into the DC family (30, 31). One of the most important findings of these early studies on LC was that DC exist in two phenotypically and functionally distinct states: as immature cells that are highly phagocytic and specialized to take up and process antigen, and as mature cells dedicated to identify and stimulate rare antigen-specific naïve T cells in secondary lymphoid organs (Figure 1). This ­observation is directly linked to another unique function of DC, namely, their migration via afferent lymphatics into the T cell areas of secondary lymphoid organs (19). In fact, in early mixed lymphocyte reaction (MLR) experiments DC turned out to be about 100-fold more efficient at inducing naive T cell proliferation than macrophages (32–34), which also do not travel from peripheral tissues to local LN (35). Owing to their easy accessibility and a large body of *in vitro* work, which almost inevitably triggers LC functional maturation, much of what we know today about the role of DC as most potent inducers of T cell immune responses stems from studying LC biology. Hence, for a long time LC were considered prototypic immunogenic DC for which Wilson and Villadangos later coined the term “*LC paradigm*” (36) and dermal DC were largely overlooked.

**Figure 3:**
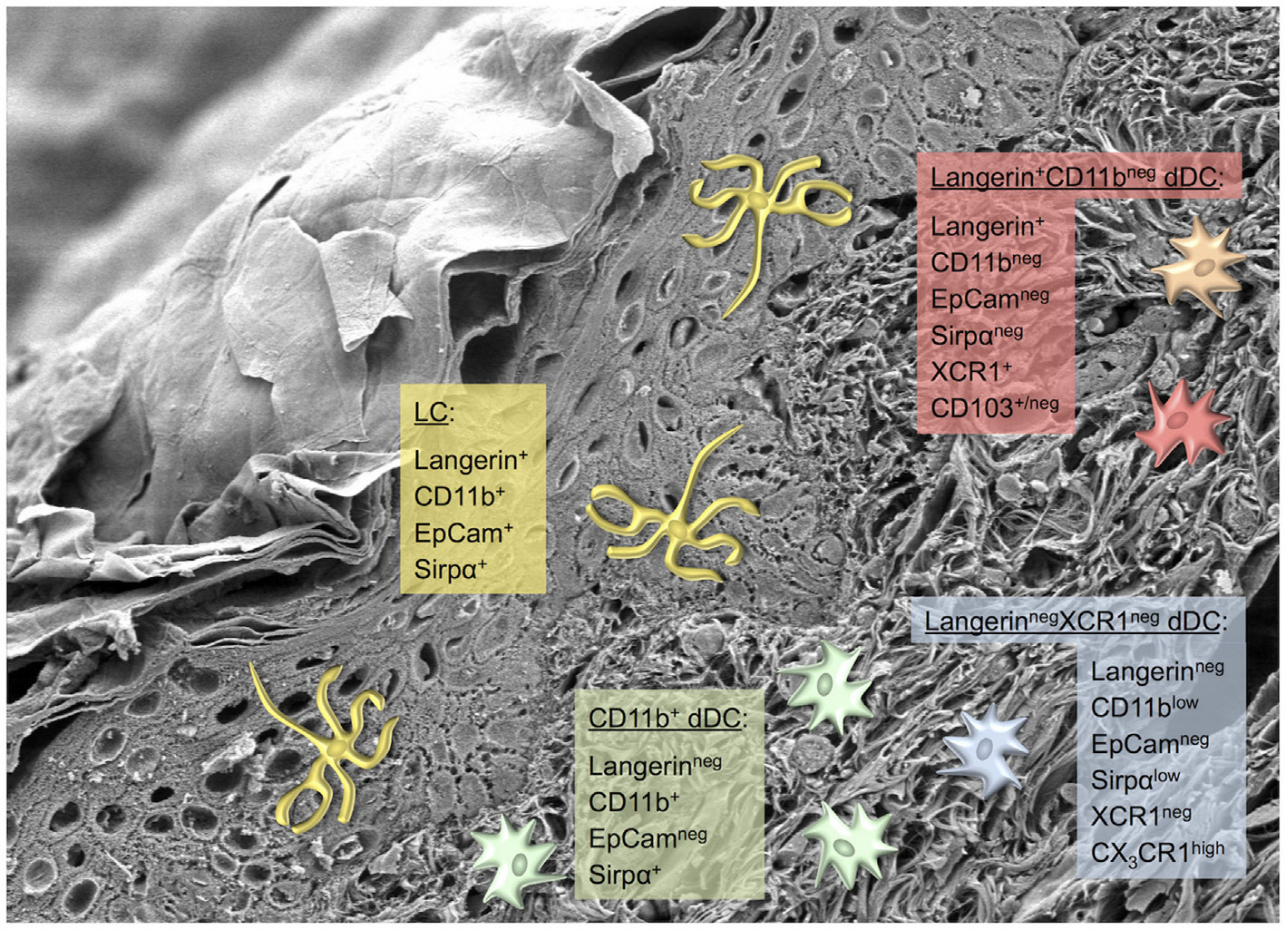
**Skin dendritic cell subsets in mice**. Scanning electron microscopy picture of a skin section depicting several layers of keratinocytes and the collagen meshwork of the dermis (38). Photograph by courtesy of Kristian Pfaller and Patrizia Stoitzner. Phenotypically distinct murine skin-resident DC subsets are depicted, including the most commonly used markers for their identification. The color code matches the human counterparts shown in Figure 4.

This picture began to change dramatically with the identification of Langerin (CD207), a novel C-type lectin specific to LC (39–41) and the generation of anti-Langerin monoclonal antibodies (42, 43). Although originally described as a LC-specific marker, in combination with constitutive and inducible Langerin^+^ cell depletion models and a Langerin-EGFP knock-in allele (44–46), this led to the discovery of a small Langerin^+^ dermal DC subset that is ontogenetically and phenotypically distinct of epidermal LC (47–49). Largely owing to the comprehensive analysis of the Malissen lab to disentangle the complexity of the skin DC network, we can currently distinguish five distinct DC subsets in healthy mouse skin (Figure 3) (50, 51). All of these DC populations express CD11c and MHC class II (MHC-II). (*i*) LC in the epidermis as well as in the dermis – en route to skin-draining LN – can be identified as Langerin^+^CD11b^+^EpCam^+^Sirpα^+^ cells, and distinguished from CD11b^+^Sirpα^+^ dermal DC by their absence of Langerin and EpCam staining. (*ii*) CD11b^+^ DC are the most abundant subset and comprise about 65% of all dermal DC (51). (*iii* and *iv*) Langerin^+^CD11b^neg^ dermal DC, on the other hand, are unambiguously recognized by expression of the chemokine receptor XCR1, lack EpCam and Sirp1α, and can be further divided into a CD103^+^ and negative subset. Expression of XCR1 is shared by all CD11b^neg^ non-lymphoid and CD8α^+^ lymphoid tissue DC, respectively, but only XCR1^+^CD11b^neg^ DC in the dermis co-express Langerin (52, 53). Of note, in the skin surface expression of CD24 correlates with that of Langerin and can be used for the purification of viable LC and Langerin^+^ dermal DC by flow cytometry. (*v*) Finally, the dermis harbors a minor population of Langerin^neg^XCR1^neg^ double-negative DC that express low levels of CD11b and Sirpα, and are uniquely CX_3_CR1^high^ (51). These five conventional skin DC populations can be separated from dermal macrophages and monocyte-derived DC by the use of CD64 (35). In particular during inflammation, large numbers of monocytes infiltrate the skin where they differentiate into CD11b^+^Ly6C^+^CD64^+^ monocyte-derived DC that have very low or lack CD11c expression. These recently identified cells play a role mainly in activating skin-resident T cells and disappear after resolution of the inflammation (35). The functional specialization of LC and the different dermal DC populations that are present in mouse skin will be discussed below.

## Origin, Transcription Factor, and Survival Factor Requirements of Skin Dendritic Cell Populations

LC are radio-resistant cells that reside in the supra-basal layer of the epidermis, closely attached to the surrounding keratinocytes via E-cadherin containing adherens junctions. In the steady state, LC exhibit a low rate of proliferation that – unlike dermal DC – is sufficient to maintain the cells locally throughout life as has been demonstrated by parabiosis experiments in mice (54) and could also be observed in human skin of hand transplant patients (55). Only in response to inflammatory changes leading to an increased loss from the epidermis are LC replaced by blood-borne progenitors. These precursors were recruited in a CCR2-dependent way and identified to represent Ly6C^high^ monocytes that enter inflamed skin and differentiate into LC in the epidermis (56, 57). Whether these monocyte-derived LC are functionally similar and equally capable to maintain themselves *in situ* remains elusive. Recent experiments indicate that the initial wave of monocyte-derived LC reconstitution after UV radiation and contact sensitizer exposure generates only short-term LC that are transient and replaced by a second wave of steady-state precursor-derived long-term LC (58, 59). On the other hand, all dermal DC populations in healthy skin are radiosensitive, have a short lifespan, and are continuously replaced by a circulating pool of bone marrow-derived DC precursors (60).

In contrast to dermal DC that originate from DC-restricted progenitors [reviewed in Ref. (2, 60)], during ontogeny LC arise first from yolk sac-derived primitive myeloid precursors around embryonic day 18 that are largely replaced by fetal liver-derived monocytes during late embryogenesis (61). These LC precursors then acquire a DC morphology and phenotype, including CD11c and MHC-II expression immediately after birth (62), whereas Langerin expression becomes apparent only 2–3 days after birth and reaches adult levels of intensity only by 3 weeks of age (63). Moreover, between postnatal days 2 and 7 the LC undergo a massive proliferative burst (62), before reaching a typical density of about 700–1,000 LC/mm^2^ in the epidermis of adult mice (37) (Figure 2). Intriguingly, LC share this embryonic ancestry from myeloid precursors and the capacity of self-maintenance throughout life without any input from the bone marrow with brain microglia. While macrophage colony-stimulating factor 1 (M-CSF or CSF-1) is essential for the development of most tissue macrophages and partly for CD11b^+^ dermal DC (64), CSF-1R-deficient mice in addition lack both LC and microglia. Their development requires the presence of the alternative CSF-1R ligand IL-34 that is constitutively produced by keratinocytes and neurons (65, 66). Based on this unique life cycle and the shared pedigree with certain tissue macrophages, LC have recently been grouped into the same lineage as macrophages (67, 68). Although ontogenetically LC and macrophages are closely related cells, in stark contrast to the sessile tissue macrophages LC migrate to LN where they prime naïve T cells to induce regulatory or effector responses. Since migration and naïve T cell priming represent cardinal features characterizing conventional DC (Figure 1), we strongly favor to keep LC in the DC family. Reciprocally, from a semantic point of view it has to be stressed that the term “macrophage” (from Greek *makrós* “large, big” and *phagein* “eat” = “big eaters”) describes a function for which DC are certainly not specialized as has been worked out so beautifully by Ralph Steinman (69–71).

Another cytokine that has been known for a long time to be essential for LC differentiation is transforming growth factor-β1 (TGF-β1) (72). Although TGF-β1 is produced by both LC and keratinocytes, LC promote their own development through an autocrine loop of TGF-β1 secretion and signaling (73). In addition, TGF-β1 is required to maintain the network of immature LC in the epidermis (74, 75). In line with the critical role of TGF-β1 for LC development, mice lacking the TGF-β1-induced transcription factor inhibitor of DNA binding 2 (Id2), or the transcription factor Runx3 that mediates DC responses to TGF-β1 both also lack LC (76–78). Moreover, Id2^−/−^ mice have dramatically reduced numbers of lymphoid organ CD8^+^ and non-lymphoid tissue CD103^+^ DC (64).

The cytokine Flt3L is a key mediator of DC commitment during hematopoiesis (79) and injection of Flt3L into mice dramatically increased DC numbers in various tissues (80). Beyond its role in DC differentiation, Flt3L regulates the homeostatic proliferation of DC to maintain peripheral DC numbers in the steady state (81). With respect to skin DC subsets, LC are not affected by the absence of Flt3 or its ligand, whereas dermal DC were reduced in Flt3^−/−^ and Flt3L^−/−^ mice (64, 82). Granulocyte/macrophage colony-stimulating factor (GM-CSF or CSF-2) is essential for DC differentiation (83), and mice lacking either CSF-2 or its receptor display a reduction of LC and CD103^+^ dermal DC (84, 85). In the absence of macrophage colony-stimulating factor (M-CSF or CSF-1), LC numbers were halved (86), and mice that lack the M-CSF receptor (CSF-1R) have no LC and reduced CD11b^+^ dermal DC, while CD103^+^ dermal DC develop normally (56, 64).

Moreover, a number of interferon regulatory factors (IRF) and other transcription factors have been implicated in the development of different DC subsets, albeit with incomplete available information concerning their effects on LC and dermal DC. IRF2-deficient mice exhibit reduced numbers of splenic CD4^+^ DC and epidermal LC, while dermal DC subsets have not been assessed (87); IRF4^−/−^ mice harbor reduced numbers of splenic CD4^+^ DC and of migratory LN DC due to a defect in dermal DC migration, which leads to an accumulation of CD103^+^ and CD11b^+^ dermal DC in the skin (88–90); and IRF8^−/−^ mice lack splenic CD8^+^ DC and non-lymphoid tissue CD103^+^ DC, including CD103^+^ dermal DC, whereas LC are unaffected (64, 76, 91–93). In addition, IRF8 also contributes to DC function: IRF8 controls CD8^+^ DC maturation and IL-12 production (94), antigen uptake and MHC-II presentation (95), the migration of LC and dermal DC to local LN (96), and the tolerogenic function of DC by inducing the expression of indoleamine 2,3-dioxygenase (IDO) (97). Although the basic leucine zipper transcription factor ATF-like 3 (Batf3) is expressed in all conventional DC, including CD11b^+^ DC, Batf3^−/−^ mice reveal a selective deficiency of CD8^+^ and CD103^+^ DC, however, the penetrance of the CD8^+^ DC defect seems to depend on the inbred background (91, 98). The transcription factor Zbtb46 represents a negative regulator of DC activation and Zbtb46-deficient mice display no alterations in DC numbers (99, 100). Nevertheless, in Zbtb46-DTR bone marrow chimeras LC and all dermal DC subsets are depleted by the injection of diphtheria toxin (82). The deletion of the late endosomal adaptor molecule p14 (LAMTOR2) caused a gradual loss of LC from newborn mice due to increased apoptosis and a defect in homeostatic LC proliferation. This effect is partly mediated by the downregulation of TGF-β receptor II on LC (59, 101). The phenotypes of different cytokine-, growth factor-, and transcription factor-deficient mice lacking distinct DC subsets are summarized in Table 1.

**Table 1:** **Phenotypes of transcription factor and growth factor/receptor knockout mice lacking specific skin-resident dendritic cell subsets^a^**.

Transcription/growth factor/receptor knockout	Lymphoid tissue DC		Skin/non-lymphoid tissue DC			Reference
		
	CD8^+^ DC	CD8^neg^ DC	LC	CD103^+^ DC	CD11b^+^ DC	
Batf3	–	↔	↔	–	↔	(91, 92, 98)
CSF-1 (M-CSF)	↔	↔	↓	n.d.	n.d.	(86)
Csf-1R	↔	↔	–	↔	↓	(56, 64–66)
CSF-2 (GM-CSF)	↔	↔	↓	↓	↔	(83–85)
Csf-2R	↔	↔	↔	↓	↔	(84, 85)
IL-34	↔	↔	–	↔	↔	(65, 66)
Flt3	↓	↓	↔	↓	↓	(64, 79, 81, 82)
Id2	–	↔	–	–	↔	(64, 76, 78)
IRF2	↑	↓	↓	n.d.	n.d.	(87)
IRF4	↑	↓	↔	↑	↑	(76, 88–90, 93)
IRF8	–	↔	↔	–	↔	(64, 76, 91–93, 98)
LAMTOR	↔	↔	–	↓	n.d.	(59)
Runx3	↑	↓	–	n.d.	n.d.	(77)
TGF-β1	↔	↔	–	↔	↔	(72–75)
Zbtb46^b^	↔	↔	↓	↓	↓	(82, 99, 100)

*^a^– indicates an absent cell population, ↓ indicates a reduction, ↔ no change, and ↑ an increase in cell number*.

*^b^Includes data from diphtheria toxin-treated Zbtb46-DTR bone marrow chimeras*.

*n.d. = not determined*.

In conclusion, the various skin DC subsets vary in their dependency on different transcription and growth factors, which allows the manipulation of particular subsets to investigate their functional properties. Our current knowledge on the specific roles of cutaneous DC subsets in allergic and infectious skin diseases as well as in skin cancer will be discussed in the following sections.

## Functional Redundancy of Skin Dendritic Cells in Contact Hypersensitivity

Contact hypersensitivity (CHS) responses to topically applied haptens in mice represent a relevant model for allergic contact dermatitis. Following percutaneous penetration, the hapten covalently binds to host proteins thereby generating a neo-antigen that is eventually recognized by the immune system (102, 103). The emergence of CHS critically depends on the activation of hapten-specific naïve T cells in skin-draining LN during hapten sensitization, which then proliferate and differentiate into effector T cells that mediate a transient ear swelling reaction at the time of hapten challenge. In agreement with the LC paradigm, although haptens can passively drain to LN via afferent lymphatics, the induction of a productive T cell response hinges on the transport of haptenized antigens by migratory skin DC to the T cell areas of the nodes. Therefore, when the first *in vivo* LC ablation mouse models were introduced, it came as a surprise that CHS was similar (46) or reduced, but not absent (44), after inducible depletion of LC in the skin prior to hapten sensitization (Table 2). These findings suggested that LC were not essential to induce the ear swelling reaction and that dermal DC contributed to T cell activation in CHS. Moreover, LC had no role in regulating the effector T cell response as was demonstrated by comparable ear swelling following diphtheria toxin treatment after sensitization but prior to hapten challenge (46, 104).

**Table 2:** **Contact hypersensitivity reactions in mice with specific defects in skin dendritic cell subsets^a^**.

Mouse strain	Epidermal LC	Langerin^+^CD103^+^ dermal DC	Langerin^neg^CD11b^+^dermal DC	CHS	Reference
**DT inducible cell depletion systems**					
Langerin-DTR (DT days −1 to −3)	–	–	↔	↓ or ↔ (dependent on hapten conc.)	(44, 46, 47, 104, 105)
Langerin-DTR (DT days −7 to −13)	–	↓ (30%)	↔	↓ or ↔ (dependent on hapten conc.)	(47, 105)
Langerin-DTR BM → WT chimeras	↔	–	↔	↔	(106)
hLangerin-DTR	–	↔	↔	↑	(107)
**Constitutive cell deficiency**					
hLangerin-DTA	–	↔	↔	↑	(45)
LC/DC-specific TGF-βR1^−/−^	–	↔	↔	↓	(75, 108)
LC/DC-specific p14^−/−^	–	↔	↔	↓	(59)
Batf3^−/−^	↔	–	↔	↔	(91)

*^a^– indicates an absent cell population, ↓ indicates a reduction and ↔ means no change in cell number, functionality or CHS intensity, and ↑ indicates an increase in the CHS reaction*.

When the Langerin^+^ dermal DC subset was discovered in 2007 (47–49), it became clear that these initial experiments had been performed in the absence of both Langerin^+^ skin DC populations and not in the selective absence of epidermal LC as one had assumed [because all Langerin^+^ DC in the dermis were considered to be LC en route to local LN (46)]. However, in agreement with their continuous replenishment from blood-borne precursors, it turned out that following injection of diphtheria toxin the dermal Langerin^+^ DC recovered much faster, i.e., within 7–10 days, while the long-lived self-maintaining LC stayed away for a prolonged period of time, i.e., at least 2–4 weeks (44, 47, 105). Using timed diphtheria toxin treatments, this enabled researchers to induce CHS when both Langerin^+^ skin DC (administration of diphtheria toxin 1–3 days prior to hapten sensitization) or only LC (diphtheria toxin treatment 7–13 days before sensitization) were lacking. Alternatively, Langerin-DTR into wild-type bone marrow chimeras permitted the selective depletion of only Langerin^+^ dermal DC before the induction of CHS (106). From this comprehensive analysis by a number of different laboratories, it became clear that the intensity of the CHS reaction is directly correlated with the efficiency of T cell priming, as was suggested by inefficient antigen transport to draining LN in the absence of Langerin^+^ skin DC (104). Consequently, and in agreement with early dose–response studies (109), LC are required for efficient induction of CHS responses, in particular, at low hapten doses, while at higher hapten concentrations sufficient amounts of antigen can be picked up by dermal DC – both Langerin^+^ and negative – for effective elicitation of CHS in the absence of LC (37, 47, 105, 106, 110, 111). Taken together, there is overwhelming evidence indicating functional redundancy of the different skin DC subsets in CHS (Table 2).

In contrast to these inducible Langerin-DTR knock-in mouse models, which harbor physiologic numbers of LC and Langerin^+^ dermal DC until the injection of diphtheria toxin, human (h)Langerin-DTA BAC transgenic mice that constitutively lack LC throughout life mounted enhanced ear swelling responses (45). Although this observation suggested that LC may exert a down-regulatory function in CHS, the great amount of data discussed above rather support compensatory roles of the different skin DC populations during the sensitization and elicitation of CHS (37, 47, 105, 106, 110, 111). Apart from these reports, it is difficult to conceive how negative regulatory properties of LC could develop or be maintained in the highly inflammatory setting of a CHS sensitizing reaction (112). However, the reason for the discrepancy between the inducible and the constitutive LC ablation models remains elusive. On the one hand, hLangerin-DTA mice may develop increased CHS as a result of some unknown failing peripheral tolerance mechanism in the lifelong absence of LC and therefore may respond differently during hapten sensitization and/or may possess altered T cell properties (37, 111, 113). On the other hand, the Langerin^+^ dermal DC that return after the toxin treatment in Langerin-DTR mice may differ from the cells that originally developed during ontogeny (58), and which are left untouched in hLangerin-DTA mice, presumably due to differences in the transcriptional regulation of the mouse and human *langerin* promotors. Both of these hypothetical explanations seem unlikely, however, because all transgenic mouse strains that constitutively lack LC (or Langerin^+^ dermal DC) as a result of varying genetic defects, i.e., independently of the diphtheria toxin/DTR system, and that have been tested in CHS mount similar or attenuated ear swelling reactions than LC-competent controls (Table 2) (75, 91, 101, 108). Instead, hLangerin-DTA mice may develop aggravated CHS due to changes in the homeostasis of dermal DC populations, i.e., an increased number of CD103^+^ dermal DC (92), or due to unknown DNA sequences that have been introduced with the human *langerin*-containing BAC. Although speculative as well, the latter may be implied, because to date hLangerin-DTR mice generated with the same BAC construct are the only other mouse model that mount enhanced CHS responses, i.e., after acute diphtheria toxin-mediated ablation of LC (107).

In conclusion, while LC clearly have regulatory potential that may have evolved to prevent inappropriate immune activation to keratinocyte-derived antigens or by commensal skin microbiota (see below), the vast majority of the available evidence indicates that LC promote the induction of CHS reactions, but are only essential at low hapten concentrations, and that dermal DC also contribute to CHS.

## Functional Specialization of Cutaneous Dendritic Cells in Infectious Skin Disease and Homeostasis to Commensal Microbiota

One of the first observations questioning the LC paradigm was the finding that during cutaneous herpes simplex virus-1 (HSV-1) infections not epidermal LC, but instead CD8α^+^ LN-resident DC were responsible for T cell priming and induction of the anti-HSV-1 response (114). Notably, LC were still required to process and transport HSV-derived antigens to the LN, where they transferred their antigenic cargo to the CD8α^+^ LN DC for cross-presentation to naïve T cells (115). Another study using an HSV-2 infection model of the vagina also revealed that epithelial LC did not present viral antigens to LN T cells (116). In this case, submucosal CD8α^neg^ migratory DC carried the viral peptides to the LN and induced the protective T helper (Th) type-1 response to HSV-2. A key question concerning these HSV infection models remains why LC played no direct role in antigen presentation and T cell activation. Was it merely because they were infected and killed by the cytopathic herpes viruses (117, 118); essentially leaving no other option for the apoptotic LC than being taken up and cross-presented to CD8^+^ T cells by LN-resident DC (119).

This hypothesis is supported in an apoptosis-inducing vaccinia virus infection model, in which cytotoxic T cell activation was similarly taken over by CD8α^+^ LN-resident DC, i.e., after uptake and cross-presentation of apoptotic skin-derived DC. On the other hand, in a cutaneous lentiviral infection model where LC/DC stay alive, migratory skin DC are perfectly capable to present antigen to T cells in the draining LN (120). Eventually, this concept was also confirmed for the HSV model, at least for Langerin^+^CD103^+^ dermal DC (121). In contrast to the primary infection via superficial skin scarification, during reactivation of the virus from its natural reservoir in the cutaneous nerves, HSV antigen presentation to CD8^+^ T cells occurred by both Langerin^+^CD103^+^ skin DC and CD8α^+^ LN DC. LC still played a minor role in direct antigen presentation, most likely due to higher sensitivity to this cytolytic virus than dermal DC. Although this concept that antigen-carrying skin DC, in particular LC, are taken up for cross-presentation by CD8α^+^ LN-resident DC cannot be generalized (119), it was later found that Langerin^+^CD103^+^ dermal DC cross-present keratinocyte-derived antigens irrespective of the presence of epidermal LC (see below) (51). A comprehensive overview of the role of DC in primary HSV infections beyond these basic principles has been published recently (122).

LC were originally also considered to be critical for the induction of protective immunity in another infectious skin disease, namely cutaneous leishmaniasis, because they were shown to transport the parasites from the site of infection to skin-draining LN (123). This view was challenged when it was reported – at about the same time that the seminal HSV infection studies were published (114, 116) – that Langerin^neg^CD8α^neg^ presumably dermal DC, but not LC, act as principal antigen-presenting cells (APC) in experimental *Leishmania major* infection (124). Resistance to *L. major* infection and healing of the skin lesions both in mice and men critically depends on the efficient induction of a Th1/T cytotoxic (Tc) type-1 response (125, 126). Langerin-DTR mice in combination with timed diphtheria toxin treatments (see above) revealed that activation of *L. major*-specific CD8^+^ T cells is significantly reduced during the early phase of the immune response following depletion of Langerin^+^ DC, without affecting the CD4^+^ T cell response and clearance of the infection (127). This demonstrated that Langerin^neg^ dermal DC were indeed essential for effective priming of CD4^+^ Th1 cells, whereas Langerin^+^ dermal DC were involved in early priming of CD8^+^ Tc1 cells.

Moreover, formation of CD4^+^ follicular helper (T_FH_)/B cell conjugates is crucial for B cell differentiation and class switch recombination to generate high-affinity antibodies for host protection following infection with *L. major* parasites (128). Recently, LC were shown to promote germinal center formation and thus antibody affinity maturation in response to *Leishmania*-derived cutaneous antigens (129), although these experiments used non-physiologic high doses of parasites that might blur early events during infection. In a model of physiologic low-dose infection with *L. major* infectious-stage promastigotes (1,000 parasites), mice depleted of all Langerin^+^ DC developed smaller ear lesions, decreased parasite loads and a reduced number of CD4^+^Foxp3^+^ Treg cells, which was accompanied by increased production of interferon γ (IFNγ) (130). Of note, despite repeated administration of diphtheria toxin over a prolonged period of time (20 weeks) Langerin^+^ DC were efficiently depleted from the skin, confirming the absence of anti-diphtheria toxin neutralizing antibody formation as had previously been demonstrated (113, 131). Intriguingly, selective depletion of LC at the time of low-dose *L. major* inoculation demonstrated that the absence of LC, and not Langerin^+^ dermal DC, was responsible for the reduced Treg cell immigration and the enhanced Th1 response, resulting in attenuated disease (130). Hence, LC act as negative regulators of the anti-*Leishmania* response in mice. This may be important to prevent complete eradication of the parasites from the host, which leads to the loss of T cell memory and susceptibility to reinfection (132, 133).

*Candida albicans* is a dimorphic fungus accountable for chronic cutaneous and systemic infections in immune-compromised hosts. On the *stratum corneum* of the skin, commensal *C. albicans* grows as budding yeast, while pathogenic *C. albicans* in the dermis and internal organs exists predominantly in its filamentous form, i.e., as pseudo-hyphae (134). This yeast-to-hyphae transition during epidermal invasion is required for both virulence and the generation of protective Th17 responses to cutaneous *C. albicans* (134, 135). On the other hand, systemic fungal immunity is achieved by innate immune mechanisms regulated by IL-17-mediated licensing of NK cells to promote the fungicidal activity of neutrophils (136).

Taking advantage of a superficial skin infection model that does not bypass the epidermis in combination with LC-deficient hLangerin-DTA mice, it was demonstrated that LC are essential for the induction of antigen-specific Th17, but not cytotoxic T lymphocyte (CTL) responses (137). Somewhat inconsistent, despite reduced IL-17 and similar IFNγ responses in the absence of LC, hLangerin-DTA mice mounted significantly increased DTH reactions after epicutaneous *C. albicans* infection, similar to the unique phenotype of these transgenic mice in CHS [(45) and as discussed above]. However, using human Langerin-specific antibodies for targeted antigen delivery to LC in hLangerin-DTR mice (not treated with diphtheria toxin), LC were also found to be sufficient for inducing Th17 cell differentiation.

By contrast, Langerin^+^ dermal DC promoted antigen-specific Th1 and efficiently cross-presented fungal antigens to activate CTL responses. At the same time, Langerin^+^ dermal DC suppressed the ability of LC to drive the generation of Th17 cells (137). A follow-up study indicated that infection with *C. albicans* yeast but not pseudo-hyphae was capable of inducing Th17 responses through a mechanism that required Dectin-1 ligation on LC and, as a consequence, LC-derived IL-6 (138). In the dermis, absent Dectin-1 engagement by *C. albicans* pseudo-hyphae prevents Th17 induction by CD11b^+^ dermal DC. Moreover, Th17 cells were found to provide protection against secondary cutaneous infection whereas Th1 cells were protective against systemic reinfection (138). Together these elegant studies established that distinct and opposing Th cell responses are determined by a combination of differences in *C. albicans* morphology and functional specialization of skin-resident DC subsets.

Beyond the functional specialization of skin DC subsets to deal with particular pathogens, there is accumulating evidence that the interactions between the resident skin microbiota and DC autonomously shape tissue homeostasis and local immunity (139). Skin tissue of mice housed under specific pathogen-free (SPF) conditions harbors Foxp3^+^ Treg as well as αβ^+^ and γδ^+^ T cells with the potential to produce IFNγ and/or IL-17A, respectively. Microbial products from skin commensals tightly regulate this balance between Treg and effector T cells as was indicated by the increase in Treg and the reduction in IFNγ and IL-17A producing T cells in germfree mice lacking microbial products from their skin (139). Consequently, protective immunity against *L. major* is severely impaired in germfree mice, as is disease-associated pathology. Intriguingly, colonization with the single skin commensal *Staphylococcus epidermidis* was sufficient to rescue cutaneous IL-17A production in germfree mice, which was dependent on IL-1 signaling in the skin. Monoassociation of germfree mice with *S. epidermidis* at the time of infection also restored immunity to *L. major* as well as pathology with increased necrosis (139). These results suggest that defects in T cell function in the steady state or during inflammation can result from an impaired IL-1-mediated dialog with skin commensals.

Moreover, colonization of the skin of SPF mice that contained a diverse microbiota with *S. epidermidis* led to an accumulation of IL-17A^+^ CD8^+^ T cells in the epidermis that enhanced innate barrier immunity by upregulation of antimicrobial peptides and limited skin invasion of the pathogen *C. albicans*. In agreement with the unique role of CD103^+^ dermal DC in antigen cross-presentation (see below), these Tc17 cells failed to develop in Batf3^−/−^ and IRF8^−/−^ (see Table 1), while the IL-17A secreting CD8^+^ T cells developed normally in constitutively LC-deficient hLangerin-DTA mice (92). Furthermore, CD11b^+^ dermal DC were required to promote the induction and/or maintenance of Tc17 cells through their capacity to produce IL-1 in response to *S. epidermidis* colonization of the skin. In conclusion, these findings reveal that the skin immune system is highly dynamic and can be readily reshaped by the coordinated action of the different skin DC subsets upon encounter of defined commensals (92).

Hence, in agreement with the extended *LC paradigm* (Figure 1), LC exhibit a great degree of functional plasticity and become tolerogenic or immunogenic depending on the nature of the invading pathogen they encounter in the skin.

## Cross-Presentation by Skin Dendritic Cells: Question Finally Answered?

For developing immunotherapeutic approaches against cancer, one prerequisite is to understand how the various skin DC subsets induce CTL responses. There has been a long-standing debate on the ability of LC to cross-present exogenous antigen to CD8^+^ T cells (37). The start of this debate was the report that LC are dispensable for the induction of cytotoxic T cell responses against skin infection with herpes virus (see above) (114), which was later confirmed for vaccinia virus (140). Subsequent work clarified that cytopathic viruses induce apoptosis in LC rendering them sole transporters of antigen. As a consequence these cells are no longer capable of directly inducing T cell responses, however, LN-resident DC and other skin DC subsets, such as Langerin^+^ dermal DC, can step in and cross-present antigen to CD8^+^ T cells (115, 141, 142).

The debate was further fueled by studies on cross-presentation of self-antigen. For this approach, transgenic mice overexpressing ovalbumin protein in an inducible or constitutive way under control of the keratinocyte-specific K5- or K14-promoter in the skin were employed (143–145). Now it was possible to examine cross-presentation of self-antigen by the various skin DC subsets in the steady state and inflammation. Early studies demonstrated that Langerin^+^ cells can cross-present ovalbumin to antigen-specific CD8^+^ T cells (26, 146, 147). This cross-presentation ability was not necessarily restricted to Langerin^+^ dermal DC, since LC purified from trypsinized epidermis and migratory LC from epidermal explants also efficiently cross-presented ovalbumin to CD8^+^ T cells *in vitro* (144, 148). Chimeric mice in which antigen cross-presentation was restricted to LC proved that LC are able to cross-present self-antigen also *in vivo*. Interestingly, cross-presentation by Langerin^+^ skin DC led to tolerance induction through deletion of antigen-specific CD8^+^ T cells (26). After the discovery of Langerin^+^ dermal DC, it came as a big surprise, when studies using K5-ovalbumin transgenic mice established that Langerin^+^ dermal DC are the sole cross-presenters of keratinocyte-derived antigen (51, 121). The localization of Langerin^+^ dermal DC adjacent to hair follicles where K5^+^ keratinocytes are present explained how this DC subset gains access to an epidermal antigen (49, 149). Moreover, in human skin keratinocyte-derived keratin bodies were found in the dermis (150). The discrepancy to the earlier studies, proving that LC can cross-present antigen, may be due to the low migratory capacity of LC in the steady state, which ensures that the LC network stays intact until inflammation causes accelerated emigration of LC to LN (21, 43, 151). Indeed, the turnover of LC in the skin is much lower than that of dermal DC as demonstrated by BrdU incorporation assays (51). The migration of all skin DC populations increases dramatically in an inflammatory setting, though with different kinetics, so that dermal DC arrive in LN much earlier than LC (46, 152). Hence, it would be interesting to investigate the cross-presentation of skin-derived antigen in an inflammatory setting at different time points after the onset of inflammation. Aside from this, most of the studies performed to date used transgenic mice overexpressing the model antigen ovalbumin in keratinocytes. Because of the high-affinity T cell receptor binding and very strong responsiveness of ovalbumin-specific CD8^+^ T cells, these findings might not reflect what happens in real life (37). Thus, these studies need to be confirmed in a more physiological setting investigating the cross-presentation of genuine self-antigens in the skin.

For the development of immunotherapeutic strategies exploiting skin DC, exogenous antigen needs to be delivered through the skin (see below). Studies on skin immunization added more issues to the controversy whether LC can cross-present exogenous antigen. First of all, LC can induce CTL when they are loaded with soluble ovalbumin *in vitro* and co-cultured with CD8^+^ T cells (148). Most importantly, topical application of ovalbumin onto the skin by either epicutaneous immunization (153) or by dissolving micro-needles (154) confirmed that Langerin^+^ DC are involved in cross-priming of CD8^+^ T cells and that LC are superior to Langerin^+^ dermal DC, in particular, when the antigen is encapsulated in nanoparticles (154). In line with this, antibody-mediated targeting of the model antigen ovalbumin to Langerin^+^ cells by intradermal injection proved that both, LC and Langerin^+^ dermal DC, can cross-present antigen to CD8^+^ T cells *in vivo* (155).

Finally, to answer the question asked above, yes, both LC and Langerin^+^ dermal DC in the skin can cross-present exogenous antigen to CD8^+^ T cells *in vitro* and *in vivo*. We would like to emphasize that cross-presentation and cross-priming must not be equated. There is the very likely possibility that the various skin DC subsets induce different functional outcomes in CD8^+^ T cell differentiation as exemplified in a recent report. Despite initial CD8^+^ T cell proliferation induced by LC and Langerin^+^ dermal DC after loading them *in situ* with protein antigen (proving cross-presentation), LC did not cross-prime T cells but rather induced cross-tolerance. By contrast, Langerin^+^ dermal DC promoted cytotoxicity, indicating that they indeed cross-primed the T cells (155). Thus, we need to better understand the differential contributions of the various skin DC subsets in CD8^+^ T cell activation leading to either CTL differentiation or tolerance induction. This knowledge is indispensable for the future development of DC-based immunotherapy of cancer.

## Skin Dendritic Cells in Cancer

Novel immunotherapeutic strategies to vaccinate through the skin are a promising area of research for the future development of anti-cancer therapies. The rationale behind this approach comes from reports on the involvement of DC in tumor immunity and their outstanding potential in promoting T cell responses. The presence of DC has been reported in many different tumors, however, their specific role in tumor immunity is still incompletely understood (156, 157). Aside from this, tumors also strongly impair DC function and actively prevent efficient immunosurveillance by DC (158). With respect to cutaneous cancer, such as squamous cell carcinoma (SCC), basal cell carcinoma (BCC), and melanoma, several reports indicate that the numbers and function of skin DC are affected by tumor growth.

So far few studies attempted to analyze the specific role of skin DC present in cutaneous tumors. Non-melanoma skin cancer, such as SCC and BCC, are tumors of basal keratinocytes, making it very likely that LC are the first APC getting in contact with transformed cells. Two studies used patient samples from SCC to investigate LC and DC in regard to numbers, phenotype, and T cell stimulatory capacity. In the first study, the numbers of LC in the SCC tumor lesions were decreased as compared to healthy epidermis. Less myeloid cells, including dermal DC, were found around tumor nests than in normal skin. Tumor-associated myeloid DC were poor stimulators of allogeneic T cells despite displaying an activated phenotype (159, 160). The second study demonstrated that tumor-infiltrating LC are more activated and induced higher CD4^+^ and CD8^+^ allogeneic T cell proliferation as well as IFNγ production than LC from adjacent healthy skin (161). Thus, LC and myeloid DC found in human SCC samples display an activated phenotype, but only LC induce allogeneic T cell responses. Although suggestive, these studies do not allow any conclusion on the functional ability of these DC subsets to promote tumor immunity to non-melanoma skin cancer since tumor-specific T cell responses were not investigated. However, unhindered tumor growth despite the activation of LC/DC indicates that the immunosuppressive milieu in SCC tumors, which contains high concentrations of TGF-β1 counteracts successful tumor immunity (159).

Another study used a murine model of chemically induced SCC to investigate the role of LC during tumor development (162). Chemical carcinogenesis was induced by application of the carcinogen 7,12-dimethylbenz(a)anthracene (DMBA) that causes Hras mutations, followed by the tumor-promoting agent 12-O-tetra-decanoyl-phorbol-13-acetate (TPA) leading to development of papilloma and subsequent SCC. The observation that hLangerin-DTA mice, lacking LC throughout their lifetime, are completely protected from tumor development is somehow surprising, but may be related to the intrinsically enhanced elicitation of adaptive immune responses, i.e., in CHS and DTH reactions, in this as opposed to other LC-deficient mouse models (see above). However, the authors propose the interesting concept that LC mediate the metabolic conversion of DMBA to its mutagenic metabolite that in turn leads to DNA damage and carcinogenesis. They further suggest that compared to keratinocytes LC express higher levels of CYP1B1, an enzyme of the cytochrome P-450 family responsible for the mutagenic metabolism of DMBA (162). How the metabolite is transferred from LC to keratinocytes to exert its DNA-damaging function was not investigated. Moreover, these data are difficult to reconcile with the fact that keratinocytes themselves express all required enzymes for DMBA metabolism (163, 164). In a follow-up paper, the authors demonstrated that LC exert pro-carcinogenic effects also independently of the enzyme CYP1B1 (165), possibly by aryl hydrocarbon receptor-mediated transcription of other CYP enzymes that trigger DNA damage (166).

In addition, LC played an important role in the progression of tumors by affecting the hyperproliferation of keratinocytes in the DMBA-induced SCC model (165) as well as in UVB-induced SCC (167), likely by augmenting IL-22 production by keratinocytes. Notably, this direct carcinogenic role of LC may be amplified by LC-driven immunosuppression, which is the induction of antigen-specific Treg cells upon UV radiation exposure (166, 168). It will be interesting to compare the growth of chemically and UVB-induced SCC in the hLangerin-DTA mouse model (165, 167) to one of the inducible Langerin-DTR knock-in mice (44, 46), since the constitutive absence of LC in hLangerin-DTA mice may have a distinct effect on the development of the skin immune system (see above). Intriguingly, a recent report revealed an unaltered expression profile of cytochrome P-450 enzymes in the absence of LC and Langerin^+^ dermal DC upon DMBA application in Langerin-DTR mice (169).

**Figure 4:**
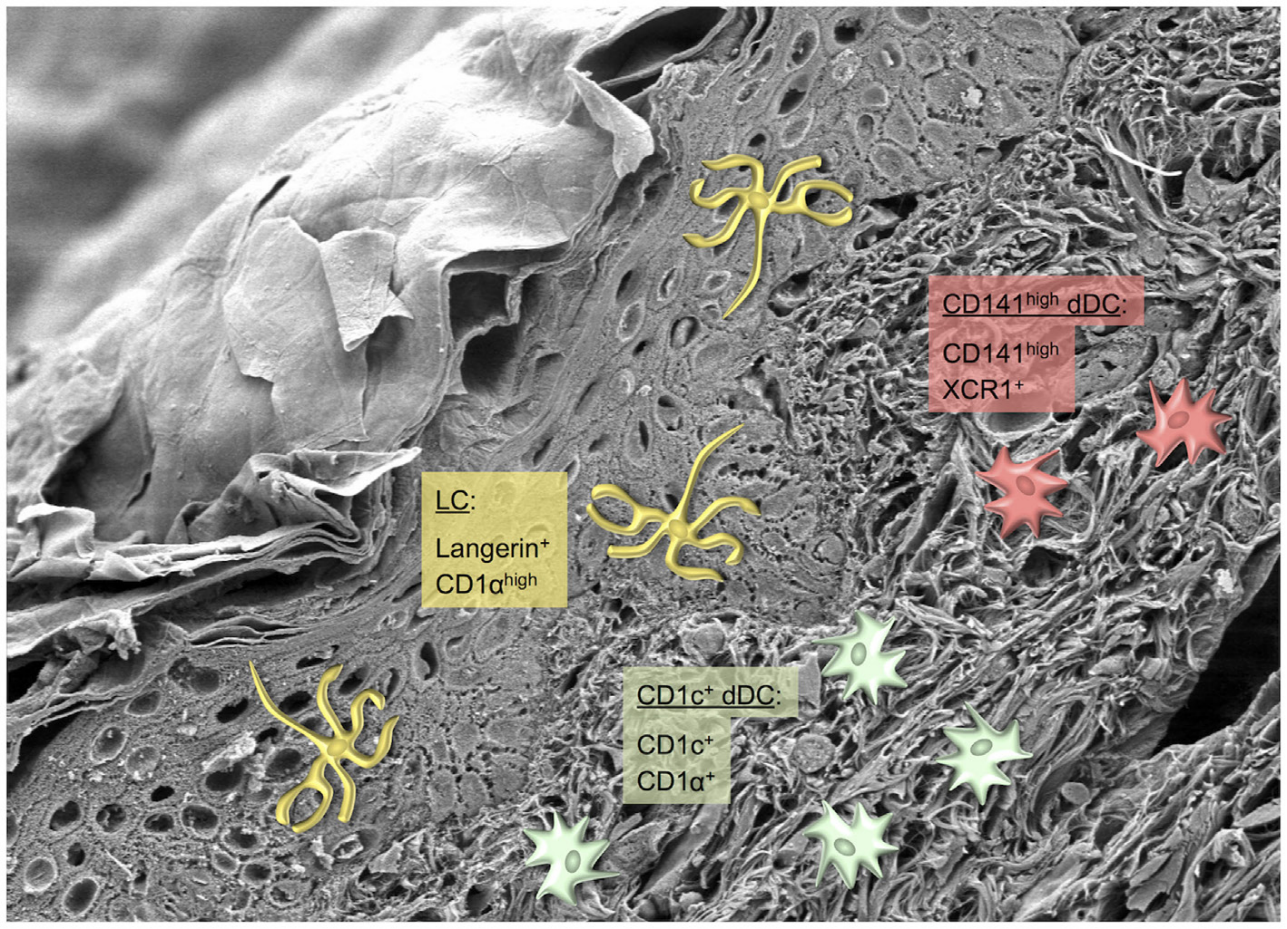
**Dendritic cell subsets in human skin**. Scanning electron microscopy picture of a section through the skin depicting several layers of keratinocytes and the collagen meshwork of the dermis (38). Photograph by courtesy of Kristian Pfaller and Patrizia Stoitzner. Phenotypically distinct human skin-resident DC subsets are described by their most prominent markers. The color code represents the murine counterparts shown in Figure 3.

Similar as in SCC and BCC, the role of the different skin DC in the immunosurveillance of melanoma is incompletely understood. Over 20 years ago first reports described decreased numbers of LC above invasive human melanoma (170, 171). In line with these findings, the presence of transplantable tumors lowered the number and impaired the migration of LC from murine skin (172). The types and relative proportions of tumor-infiltrating DC in melanoma have not been determined so far, such that information on the functional potential of distinct skin DC subsets to control melanoma is lacking. For instance, in melanoma the accumulation of mature DC of unknown origin in draining LN metastases was associated with the expansion of antigen-specific cytotoxic T cells (173). A recent effort to identify the various myeloid cell types within a transplantable murine melanoma model demonstrated that the CD103^+^ DC subset, which most likely includes Langerin^+^ dermal DC, was superior over CD11b^+^CD103^neg^ DC in cross-presenting tumor antigens (174). Future studies using multi-color flow cytometry including a comprehensive panel of markers to discriminate individual DC subsets (Figures 3 and 4) will be required to obtain a detailed picture on the involvement of distinct skin DC in tumor immunity (175). This knowledge will form the basis for the design of novel and for the improvement of existing immunotherapies harnessing the potential of skin DC for the immunotherapy of cancer.

## Harnessing Skin Dendritic Cells as Targets for Immunotherapy of Cancer

From the time of the experiments of William Coley in the early 1900s immunotherapy of cancer was in the minds of immunologists (176), though it played a rather marginal role. Next to T cells, cytokines and antibodies, DC became promising targets for immunotherapy of cancer owing to the pioneering work by the late Ralph Steinman, who received the 2011 Nobel Prize in Physiology or Medicine for “his discovery of the DC and its role in adaptive immunity” (177). His ultimate goal was to harness the outstanding immunogenic properties of DC for immunotherapy and thereby “taking dendritic cells into medicine” (178). Since the first report of treatment of a B cell lymphoma with antigen-pulsed DC from blood (179), many clinical and basic research centers have been working to improve the efficacy of DC-based strategies to treat cancer patients. The first adoptive DC therapy for cancer (Provenge™, a tumor antigen-pulsed cell suspension containing DC) was approved by the FDA in 2010, and this constituted a milestone in the development of cellular therapies. A parallel development in cancer immunotherapy occurred over the past few years when the so-called “checkpoint inhibitors” were introduced into clinical practice. These antibodies against CTLA-4, PD-1, or PD-L1 (and presumably other inhibitory mediators in the future) switch off down-regulatory signaling pathways in T cells. They are therefore able to powerfully unleash anti-cancer immunity that exists in patients but is obviously insufficient or suppressed by various mechanisms (180). Clinical responses in patients are impressive, but so are side effects (autoimmunity), particularly with the anti-CTLA-4 antibodies. Still, these clinical observations have earned this therapeutic approach the title of “Breakthrough of the Year” in 2013 by *Science* (181).

In spite of these encouraging developments targeting T cells, the potential of DC-based therapies remains high for several reasons. (*i*) Only a variable part of patients treated by checkpoint blockade responds to the treatment (180, 182, 183). (*ii*) Even though undesired autoimmunity can be clinically managed, it would be advantageous to avoid or minimize it from the beginning. (*iii*) Most importantly, checkpoint blockade can only boost those cancer-specific T cells that are already preexisting in the patient. By contrast, DC therapy would be able to generate *de novo* immune responses (182, 183). Such responses would be desired against neo-antigens (“private mutations”) in patients’ tumors (182, 184). DC-induced T cell responses against such mutated tumor antigens would additionally lack autoimmune danger.

The *ex vivo* generation of DC for therapy is laborious, nevertheless, therapy with tumor antigen-pulsed autologous DC proved to be safe and effective, though not curative, in patients with solid tumors (185). The continuing importance of DC therapy is highlighted by recent publications, indicating persuasive ways of improving DC vaccines. For instance, DC were loaded with peptides derived from neo-antigens identified from the patient’s own tumor material, instead of the commonly used peptides from overexpressed self-antigens. This strategy augments T cell responses by broadening the antigenic diversity of the anti-tumor response in the absence of autoimmunity (186). Another approach is the conditioning of the injection site with a potent recall antigen, such as tetanus toxoid, TLR ligands, or cytokines. The pretreatment of the skin site with antigen or danger signals improved the migration of adoptively transferred DC and boosted T cell responses in tumor-bearing mice (187–189). Moreover, the co-administration of tumor-binding allogeneic antibodies enhanced the internalization of tumor antigens by DC and dramatically increased therapy outcome in murine tumor models (190). Furthermore, the choice of DC subset could enhance the efficacy of DC therapy as has been demonstrated for LC-like cells generated from CD34^+^ precursor cells that were able to overcome tolerance to differentiation antigens commonly overexpressed in cancer patients and used for vaccination (191).

Yet another imaginative approach that has been pioneered many years ago in Ralph Steinman’s laboratory (192, 193) is the use of antibody-mediated antigen targeting constructs to specifically deliver antigenic peptides or proteins to DC *in situ*. The basic idea is to target the antigen of interest to endocytic receptors specific for DC (subsets) resident in the skin or lymphatic organs to enable them to efficiently incorporate antigen for presentation to T cells. This approach would be much less laborious than the *ex vivo* generation of DC from patients’ monocytes and their subsequent loading with tumor antigens. Intriguingly, antigen delivered without adjuvant can induce tolerance, whereas concomitant administration of TLR ligands and agonistic anti-CD40 antibody causes strong induction of CD4^+^ and CD8^+^ T cell responses (192, 194). In addition, this approach allows delivering antigen into the various skin DC subsets by aiming at different lectin receptors (195, 196). The most interesting candidate receptors for these *in vivo* antigen targeting approaches are C-type lectins, which are expressed by skin DC, e.g., DEC-205, Langerin, and Dectin-1 (197–199). Specific delivery of the model antigen ovalbumin into Langerin^+^ cells proved that both LC and Langerin^+^ dermal DC can cross-present antigen to CD8^+^ T cells, however, only the dermal DC promoted the development of cytotoxicity while LC induced tolerance (155). Targeting antigens to DEC-205^+^ DC led to tumor control or even eradication in murine tumor models, albeit the relative roles of skin DC and LN-resident DC were not investigated in these studies (194, 200, 201). The chemokine receptor XCR1, which is preferentially expressed on Langerin^+^ dermal DC (52, 53) (Figure 3), is another promising target due to the fact that this DC subset excels in inducing cytotoxicity in CD8^+^ T cells (155). Immunization with the chemokine XCL1 conjugated to ovalbumin protein intravenously or through skin pre-treated with a laser to form pores led to CD4^+^ and CD8^+^ T cell activation and inhibited the growth of transplanted tumors in mice (202, 203). First clinical trials have been initiated and so far demonstrated induction of some humoral and cellular immunity by targeting the tumor antigen NY-ESO-1 conjugated to DEC-205 antibody into DC of patients with various solid tumors (204). These data demonstrate the proof-of-concept of the targeting approach in human cancer, but they also call for intense further study to substantially improve this strategy.

Immunization strategies through the skin are very attractive for their easiness of use and, ultimately, the possibility of self-medication in case of topical treatment. Several approaches have been developed such as epicutaneous immunization, micro-needles, laserporation, or powder injection (205). The epicutaneous approach allows to topically apply antigens in protein and peptide form onto the skin (206). The disruption of the skin barrier and the addition of an adjuvant proved to be essential to elicit powerful cytotoxic T cell responses that inhibit tumor growth (153, 207, 208). The involvement of Langerin^+^ skin DC in CD8^+^ T cell responses was confirmed in experiments with Langerin-DTR mice in a tumor setting. In line with findings in the CHS model (see above), the antigen dose of ovalbumin protein determined which skin DC subset presented the tumor antigen. The inhibition of tumor growth by epicutaneous immunization with low-dose antigen was completely abrogated in the Langerin-DTR mice depleted for Langerin^+^ DC, whereas application of a higher dose of antigen still partly inhibited tumor growth even in the absence of Langerin^+^ DC (153). This supports the notion of the high plasticity of skin DC subsets. The epicutaneous immunization approach has already been clinically tested and proved to be promising for the treatment of cancer and infection (209, 210).

Another very elegant approach is the application of dissolving micro-needles that allow delivery of antigens right into the tissue where skin DC are located. This strategy has been successfully used to vaccinate against influenza (211) and to treat tumors, at least in murine models (154). The latter study demonstrated that LC can be superior to dermal DC in cross-presentation of antigen delivered with micro-needles into the skin. Interestingly, the nature of the antigen determined which skin DC subset induced CD8^+^ T cell proliferation, in that encapsulated antigen was preferentially cross-presented by LC whereas soluble antigen required Langerin^+^ dermal DC for CD8^+^ T cell activation. The CD4^+^ T cell response was promoted by all skin DC subsets, however, LC dominated the induction of Th1 and Th17 responses (154).

For the future, it will be worthwhile to investigate the potential of the various cutaneous DC subsets in skin immunization and translate the findings from murine tumor models to the patient situation. Undoubtedly, skin DC are critically involved in surveying the skin in order to prevent tumor growth and clearly fulfill an immunogenic role during vaccination against cancer. Yet their precise roles are still unclear and need to be clarified before we will arrive at urgently needed more effective DC-based treatment options for cancer.

## Human Skin Dendritic Cell Subsets

The human and murine skin DC network seems to be highly conserved between the two species. While this justifies *in vivo* experiments in mice to gain mechanistic insight into the functional specialization of cutaneous DC subsets in regulating immunity and tolerance, we need to identify the human counterparts of the murine DC subtypes in order to translate this knowledge to treating patients. In the recent years, human skin DC subsets were better defined and found to be homologous to murine DC (Figure 4) (212–214). In humans, the epidermis contains LC expressing Langerin and high levels of CD1a, whereas in the dermis three subsets of dermal DC can be distinguished (37, 215). The largest population is represented by the CD1c^+^CD1a^+^ dermal DC, which correspond to the murine CD11b^+^ dermal DC (216, 217). The smallest subset of DC in human dermis is characterized by high expression of CD141 and XCR1 and is homologous to the murine Langerin^+^CD103^+^ dermal DC, which also express XCR1 (214, 218). Very recently evidence for yet another small subset of (weakly) Langerin^+^CD1c^+^ dermal DC was presented, highlighting that similar to mice Langerin expression may not be strictly confined to LC in human skin (219). The CD14^+^ dermal DC are monocyte-derived cells that are transcriptionally aligned rather to monocytes/macrophages than to DC (216). A corresponding tissue-resident DC subset with the phenotype CD11b^+^Ly6C^−^CD64^lo-hi^ has been described in murine dermis (35).

In regard to functional aspects, both human LC and CD1c^+^ dermal DC can polarize Th1 and Th2 responses (220), depending on the cytokine milieu in the skin (221), and cross-present exogenous antigen to CD8^+^ T cells (215). However, the recently discovered CD141^hi^ dermal DC excel in cross-priming of CD8^+^ T cells comparable to murine Langerin^+^ dermal DC (214). There still exists some controversy in the field on the suitability of XCR1 and CD141 as markers for cross-presenting DC since both molecules can also be expressed by some CD1a^+^ and CD14^+^ dermal DC (218, 222, 223). Importantly, the methods used for the preparation of skin DC differ strongly between the many studies published. Notably, DC isolated from skin tissue by enzymatic digestion or derived from skin explant culture after emigration from the tissue (“crawl-outs”) represent immature and mature DC, respectively. This has major influence on their phenotype/function and the presence/absence of cell surface markers. One example is CD141, which is differently expressed on freshly isolated dermal DC and migratory dermal DC. While CD141 is upregulated on CD14^+^ dermal DC upon emigration from skin explants as compared to DC enzymatically isolated from skin, its expression on CD141^high^ CD14^neg^ dermal DC remains unaltered upon migration (214, 223). Humoral immunity is mainly modulated by CD14^+^ dermal DC since they can activate the differentiation of T_FH_ cells (220). Moreover, CD14^+^ dermal DC support memory T cell activation, most likely *in situ* in the skin, but they are poor stimulators of naïve T cells (216). During inflammation, several inflammatory DC subsets, e.g., inflammatory dendritic epidermal cells (IDEC), 6-sulfoLacNAc^+^ (slan) DC, and TNF-α/iNOS-producing (Tip) DC, are recruited to the skin and have a strong impact on the course of inflammatory skin diseases such as psoriasis and atopic dermatitis (215, 224, 225).

Despite the high degree of homology between mouse and human skin DC, functional disparities do exist. Some examples are listed here: (*i*) For instance, with the help of IL-15 released into the immunological synapse, human LC cross-prime cytotoxic CD8^+^ T cell responses (226, 227). As a consequence human LC are able to break tolerance to self-antigens and stimulate cytotoxic T cell responses (191). So far hardly any information on the production of IL-15 by murine skin DC is available. (*ii*) CD70, a molecule involved in DC–T cell interaction and important for activation of CD8^+^ T cells and IFNγ production (228), is highly expressed by human LC (229, 230), whereas murine LC show very low levels of CD70 even after stimulation (155). These functional disparities between murine and human LC are supported by recently published gene transcription profiles of human skin DC subsets, indicating that human LC are more closely related to murine Langerin^+^ dermal DC than to murine LC (222). (*iii*) Another example for functional differences stems from the fact that murine LC, despite initial cross-presentation and induction of CD8^+^ T cell proliferation (148), fail to stimulate cytotoxicity in CD8^+^ T cells and instead induce cross-tolerance (155). Notably, the TLR ligands used in the latter study specifically activate Langerin^+^ dermal DC (231), thus we need to evaluate the potential of LC in cross-priming with TLR ligands suitable for their activation. The knowledge on functional properties of the various murine and human skin DC subsets is of eminent importance when we envisage novel immunotherapeutic approaches that need first to be tested in preclinical murine studies before they can be translated into the clinics.

## Conclusion

The skin, as one of the barrier tissues to the environment, harbors particular challenges to the resident DC network. While the cells continuously probe their surroundings for invading pathogens, DC have to discriminate harmless from dangerous microbes, prevent inappropriate immune reactions against self-antigens, and limit collateral tissue damage once inflammation occurs during protective immune responses. The original concept that immature DC confer tolerance and mature DC initiate immunity (232) turned out to be too simplistic and was further developed to give way to the hypothesis of functional specialization of particular DC subsets (233), including distinct pathway(s) of tolerogenic DC maturation (14, 17, 18). Through major advancements in multi-color flow cytometry, next-generation transcriptomics and proteomics, and the generation of novel cell type-specific gene targeting, cell labeling, and cell ablation mouse models, we can now dissect an increasing number of phenotypically distinct DC subsets in the skin (as well as other barrier tissues) and are beginning to unravel their functional heterogeneity (1, 3, 37). From these exciting *in vivo* experiments, it is becoming increasingly clear that specific DC subsets indeed exert specialized functions, but that this “*division of labor*” is not intrinsically defined by or fixed within one type of DC and rather determined by the signals the DC receive from their micro-environment (234). For example, LC induce Treg during *L. major* and Th17 cells upon *C. albicans* infection (130, 137), Th2 cells under the influence of pro-allergic TSLP (221) and strong Th1 responses when conditioned by a tumor environment (161). Thus, despite their context-dependent specialization, overall DC subsets display an amazing functional plasticity (“*multitasking*”). The future challenge lies in better understanding (*i*) the unique contribution of the different DC subsets to particular chronic inflammatory diseases and (*ii*) the context-dependent signals that control the function of individual DC subsets in a given disease state. This knowledge will be vital to harness (skin) DC subsets for the treatment of human diseases ranging from allergy and autoimmunity to chronic infections and cancer.

## Conflict of Interest Statement

The authors declare that the research was conducted in the absence of any commercial or financial relationships that could be construed as a potential conflict of interest.
